# Infectious endocarditis: interdisciplinarity or dual responsibility?

**DOI:** 10.1186/1471-2334-14-S7-P37

**Published:** 2014-10-15

**Authors:** Voichița Lăzureanu, Ciprian Gandac, Teodora Moisil, Virgil Musta, Narcisa Nicolescu, Ruxandra Laza, Oana Plavitu, Alexandru Crişan

**Affiliations:** 1 Dr. Victor Babeş University of Medicine and Pharmacy, Timişoara, Romania; 2 Dr. Victor Babeş Clinical Hospital of Infectious Diseases and Pneumology, Timişoara, Romania

## Background

Infectious endocarditis remains – due to its clinical polymorphism – a disease that is sometimes late diagnosed.

## Case report

We present a man, 56 years, admitted in our department for tuberculous (TB) meningoencephalitis (history of pulmonary tuberculosis, symptoms: fever, dizziness, sleepy, difficulty in speaking and walking; in the CSF proteins=62.1 mg/dL, 17 lymphocytes/cmm, chest X-ray: nodular opacity in the upper left lobe, reticulo-nodular image bilaterally, pneumologist consult: pulmonary tuberculosis, secondary infiltrative nodular upper left lobe) with favorable evolution under treatment with: meropenem 2 g Q8h + category II 7/7 TB drugs. After 7 days from admission the blood culture tested positive for *Streptococcus gallolyticus* (BACTEC). The second cardiac examination revealed during transthoracic echography a 1.8 cm vegetation on the aortic valve. We switched to vancomycin 1g Q12h plus tuberculostatics. Cardiac surgery consult recommends 4 weeks of treatment with vancomycin and then probably surgical intervention.

## Conclusion

The laboratory has a very important role in the diagnosis of infectious diseases. A close contact with cardiologists has to be established in order to have carefully investigated patients.

## Consent

Written informed consent was obtained from the patient for publication of this Case report and any accompanying images. A copy of the written consent is available for review by the Editor of this journal.

